# Dominant follicle growth patterns and associated endocrine dynamics in anovulatory and ovulatory waves in women

**DOI:** 10.1530/RAF-22-0131

**Published:** 2023-06-21

**Authors:** Shah T Bashir, Angela R Baerwald, Melba O Gastal, Roger A Pierson, Eduardo L Gastal

**Affiliations:** 1 Animal Science, School of Agricultural Sciences, Southern Illinois University, Carbondale, Illinois, USA; 2 Department of Academic Family Medicine, College of Medicine, University of Saskatchewan, Saskatoon, Saskatchewan, Canada; 3 Department of Obstetrics and Gynecology, College of Medicine, University of Saskatchewan, Royal University Hospital, Saskatoon, Saskatchewan, Canada

**Keywords:** ovulatory follicle, spontaneous cycles, anovulatory and ovulatory follicular waves, gonadotropins, steroids

## Abstract

Growth patterns and associated endocrine profiles were compared between dominant anovulatory (ADF) and ovulatory follicles (OvF) developing from different waves within and between menstrual cycles in women. Follicular mapping profiles of 49 healthy women of reproductive age and blood samples were obtained every 1–3 days during one interovulatory interval. Sixty-three dominant follicles were classified into wave 1 (W1ADF; *n* = 8) and wave 2 (W2ADF; *n* = 6) anovulatory follicles and wave 2 (W2OvF; *n* = 33) and wave 3 (W3OvF; *n* = 16) ovulatory follicles. Comparisons were made between W1ADF and W2ADF, W2ADF and W2OvF, and W2OvF and W3OvF. The waves were numbered 1, 2, or 3 based on when the waves emerged relative to the preceding ovulation. W1ADF emerged closer to the preceding ovulation, and W2ADF emerged in the late luteal or early follicular phase. The interval from emergence to maximum diameter was shorter for W2ADF than W1ADF and for W3OvF than W2OvF. Selection of W3OvF occurred at a smaller diameter compared to W2OvF. W1ADF regressed at a faster rate than W2ADF. Also, W1ADF were associated with lower mean follicle stimulating hormone (FSH) and higher mean estradiol than W2ADF. In contrast, W3OvF were associated with higher FSH and luteinizing hormone (LH) compared to W2OvF. However, W2OvF were associated with higher progesterone than W3OvF. This study contributes to the understanding of the physiologic mechanisms underlying selection of the dominant follicle, ovulation, and pathophysiology of anovulation in women, as well as optimization of ovarian stimulation protocols for assisted reproduction.

**Lay summary:**

Ovarian follicles (the fluid-filled sacs that contain the eggs) develop within 2 or 3 waves during the human menstrual cycle. Typically, only one follicle in each cycle releases an egg during ovulation. The rest of the follicles grow then regress. In this study, we characterized differences in the growth, regression, and associated hormone production between follicles that ovulated and those that did not, developing from different waves within and between menstrual cycles. Differences regarding follicle growth patterns and hormone concentrations were found when comparing different waves and whether a follicle ovulated. Results from this study improve our understanding of the processes underlying follicular growth and ovulation, which in turn may assist in improving fertility and birth control treatments.

## Introduction

Antral follicle development follows wave-like patterns characterized by two–three waves during the human menstrual cycle (Baerwald *et al.* 2003*a*,*b*). The follicle dynamic patterns observed in women are similar to those described in mares (Pierson & Ginther 1987, Ginther 1990, Gastal *et al.* 2021, Duval *et al.* 2022) and heifers (Sirois & Fortune 1988, Ginther *et al.* 1989); thus, mares and heifers (mono-ovulatory species) have been established as appropriate animal models for studying ovarian antral follicular dynamics in women (Ginther *et al.* 2004, 2005, Baerwald 2009, Gastal 2011*a*,*b*, Adams *et al.* 2012, Ginther 2012). Despite knowledge of antral follicular waves in women, the growth and the associated endocrine dynamics of dominant anovulatory (ADF) vs ovulatory follicles (OvF) developing within different waves in women are not fully understood.

Follicular dynamics in women comprises a combination of minor and major waves throughout the interovulatory interval (IOI). An IOI is the period of time from one ovulation to the subsequent ovulation. In women of reproductive age (range: 19–43 years), major ovulatory waves develop in the follicular phase (Baerwald *et al.* 2003*a*,*b*) and are preceded by one or two major or minor anovulatory waves in the same follicular phase and/or previous luteal phase. In major waves, usually a single ‘dominant’ follicle becomes morphologically selected at approximately 10 mm in diameter and then undergoes preferential growth; major waves may be ovulatory or anovulatory (Baerwald *et al.* 2003*a*,*b*, Ginther *et al.* 2004). In minor waves, the largest follicle does not undergo selection and reaches a maximum diameter that is <10 mm (Baerwald *et al.* 2003*b*). Women of reproductive age with two waves (68%) developed minor–major (85%) or major–major (15%) wave patterns throughout the IOI; women with three waves (32%) developed minor–minor–major (62%), minor–major–major (19%), or major–major–major (19%) wave patterns (Baerwald *et al.* 2003*b*). In women of advanced reproductive age (45–55 years), follicle dynamics of major waves, but not minor, have been characterized (Vanden Brink *et al.* 2013). In this age group, luteal phase-dominant follicles have been shown to emerge earlier, grow for a longer period, and reach larger diameters compared to those in women of mid-reproductive age (18–35 years; Vanden Brink *et al.* 2013). Interestingly, in one woman of advanced reproductive age, ovulation of a luteal phase-dominant follicle was documented during menses, resulting in three ovulations over the course of one cycle (Vanden Brink *et al.* 2015).

An earlier rise in preovulatory serum estradiol, luteinizing hormone (LH), and follicle stimulating hormone (FSH) has been demonstrated in women of reproductive age with two-wave cycles compared to three-wave cycles (Baerwald *et al.* 2003*b*); no differences in luteal phase progesterone were found between groups (Baerwald *et al.* 2005). Preliminary data suggest that two vs three rises in FSH may be associated with the development of two vs three follicular waves across the IOI; however, further research is required to confirm these findings (Baerwald *et al.* 2003*b*). Work is ongoing to determine whether the number of follicle waves in women is consistent across multiple menstrual cycles (Antaya & Baerwald 2020).

Early studies byBaerwald *et al.* (2003*a*, *b*) included comparisons of serum hormone concentrations in women with two vs three follicular waves across the menstrual cycle and among women with three different major/minor patterns of follicle wave dynamics. However, follicle growth dynamics and hormone production were not evaluated between ovulatory vs anovulatory follicles developing within different waves of the same cycle. Evaluating the temporal associations between reproductive hormones and growth profiles of ADF vs OvF may provide insight into the physiologic mechanisms underlying follicle growth and regression, selection of dominant follicles, ovulation, anovulation, and future oocyte competence. Information gathered from this approach may also explain why some women develop two follicular waves while others develop three waves across the cycle. We anticipate that knowledge about the hormonal regulation of ovarian follicular waves in women will be used to identify biomarkers to predict the number and types of waves that will develop. We further hope that the results of our research can be applied to the optimization of ovarian stimulation and hormonal contraceptive regimens.

The objective of the present study was to compare the intervals, growth rates, diameters, and associated systemic endocrine profiles of ADF vs OvF, developing from different types of follicular waves (i.e., W1ADF vs W2ADF, W2ADF vs W2OvF, and W2OvF vs W3OvF) within and between menstrual cycles.

## Materials and methods

### Participants

Data evaluated in the present analyses were collected from a previous study (Baerwald *et al.* 2003*a*). The novel data herein presented comprehensively compare intervals, diameters, and growth rates related to follicle emergence, deviation, ovulation, and the associated systemic endocrine profiles of ADF vs OvF developing from different waves within and between spontaneous menstrual cycles. A total of 56 women aged 28.2 ± 1.0 years (mean ± SEM; range = 19–41 years) with a history of regular menstrual cycles (range, 21–31 days long) were evaluated over one IOI. An IOI was defined as the period of time from one ovulation to the subsequent ovulation (Ginther *et al.* 2004). An IOI was chosen for evaluation to maintain consistency in data analyses and facilitate comparisons across different species. The current dataset included 49 women with ovulatory cycles (i.e., 33 IOIs with 2 follicular waves and 16 IOIs with 3 follicular waves). Seven women did not ovulate and were excluded from the analysis.

### Ethics approval

The study protocol for the original study (Bio 88-80) was approved by the Biomedical Research Ethics Board of the University of Saskatchewan, Saskatoon, SK, Canada, and the Strategic Planning and Priorities Committee of the Saskatoon Health Region. Secondary analyses were approved by the Institutional Review Board of Southern Illinois University, Carbondale, IL, USA.

### Follicle growth dynamics

The growth and regression profiles of individually identified antral follicles were retrospectively characterized following transvaginal ultrasonography of the ovaries every 1–3 days during one IOI (Pierson & Ginther 1988, Knopf *et al.* 1989, Baerwald *et al.* 2003*a*). Dominant follicle growth was characterized from the day of emergence until the follicle regressed or ovulated. Follicle diameters were evaluated on the days of emergence, deviation, maximum attained size, and ovulation. Follicle growth rates were calculated as the difference in follicle diameter between two physiologic follicular events (e.g., follicle emergence and deviation) divided by the number of days between events. Time intervals between several physiologic events were calculated.

### Definitions

The luteal phase was defined as the period between the day of the first ovulation and the first day of menses. The follicular phase was defined as the period between the first day of menses and the day preceding the subsequent ovulation. Follicle emergence was determined retrospectively to be the day before a progressively growing follicle reached a diameter of 7 mm. Deviation was defined as the day preceding the first difference in growth rates between the dominant and subordinate follicles, leading to the continued growth of the dominant follicle and regression of the subordinate follicles (Gastal *et al.* 1997, Ginther *et al.* 2004). The growth phase was defined as the interval from emergence to the maximum diameter of the dominant follicle. The static phase was defined as the interval from maximum diameter of the dominant follicle to the first day of progressive regression. The regression phase was defined as the interval from the day of ADF maximum diameter until regression to a diameter ≤7 mm.

**Figure 1:**
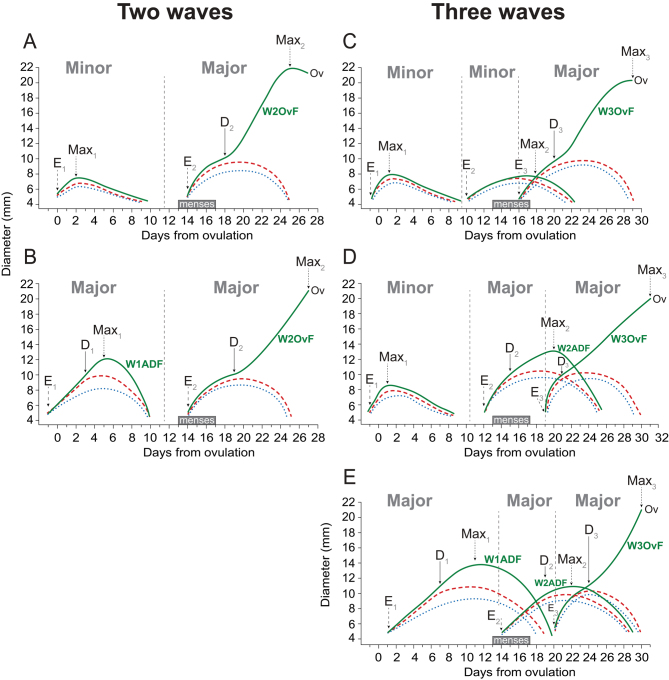
Patterns and combinations of different types of follicular waves found during the IOI/menstrual cycle in women containing the anovulatory and ovulatory follicles used in this study. To compose each wave, mean data relative to diameters and days at emergence (E; largest follicle at 7 mm), deviation (D; day preceding the first difference in growth rates between the future dominant and subordinate follicles), and maximum (Max) for the largest follicle of each wave were obtained from 50 women enrolled in the original study (Baerwald *et al.* 2003*b*). To facilitate visualization, only the three largest follicles of each wave were plotted. Small subscript numbers following an acronym (e.g., E_1_, E_2_, E_3_) reflect the end point for each sequential wave. The (A and B) two-wave and (C, D, and E) three-wave patterns of follicular waves are shown. (A) Minor wave (i.e., with the absence of a dominant follicle) preceding a major ovulatory wave (*n* = 29 women). (B) Major anovulatory wave preceding a major ovulatory wave (*n* = 5). (C) Two minor waves preceding a major ovulatory wave (*n* = 10). (D) One minor and one major anovulatory wave preceding a major ovulatory wave (*n* = 3). (E) Two major anovulatory waves preceding a major ovulatory wave (*n* = 3). The dominant follicles compared in this study and originated from different follicular wave patterns were (A and B) W2OvF (wave 2 ovulatory follicle), (B and E) W1ADF (wave 1 anovulatory dominant follicle), (C, D, and E) W3OvF (wave 3 ovulatory follicle), and (D and E) W2ADF (wave 2 anovulatory dominant follicle). Menses is indicated in each IOI. Ovulation, Ov.

Minor waves, characterized by the absence of dominant follicles (i.e., largest follicle <10 mm;Baerwald *et al.* 2003*b*), were considered in this study only to determine the number of waves within each cycle; growth and related endocrine characteristics of follicles within minor waves were not the focus of this study. The major follicular waves (i.e., with the presence a dominant follicle ≥10 mm;Baerwald *et al.* 2003*a*, *b*, Ginther *et al.* 2004) were classified and defined as follows: Wave 1 anovulatory dominant follicles (W1ADF), which emerged in the early luteal phase in women with two or three waves (*n* = 8 women); Wave 2 anovulatory dominant follicles (W2ADF), which emerged in the late luteal to early follicular phase in women with three waves (*n* = 6 women);Wave 2 ovulatory follicles (W2OvF), which emerged in the early follicular phase in women with two waves (*n* = 33 women); and Wave 3 ovulatory follicles (W3OvF), which emerged in the early–mid follicular phase in women with three waves (*n* = 16 women). Examples of follicular waves (i.e., minor and major) in women with two- or three-wave cycles as well as the association with ADF and OvF evaluated during the IOI are shown in Fig. 1.

### Blood collection and hormone analyses

Blood samples were drawn every 3 days during the IOI in a stratified manner among participants as described (Baerwald *et al.* 2003*b*, 2005). Interassay coefficients of variance were as follows: FSH (low = 8.0%, medium = 2.9%, high = 4.1%), LH (low = 6.3%, medium = 4.0%, high = 4.5%), estradiol (low = 9.8%, medium = 5.6%, high = 4.3%), and progesterone (low = 10.8%, medium = 7.0%, high = 10.8%). The assay sensitivities were 0.1 mIU/mL for FSH, 0.1 mIU/mL for LH, 15 pg/mL for estradiol, and 0.2 ng/mL for progesterone.

Hormonal data were grouped into 3**-**day intervals to obtain a relevant representation of endocrine dynamics over the IOI. The data were then centralized ±1 day to specific follicular events such as emergence, deviation, maximum diameter, and ovulation, as previously described (Ginther *et al.* 2005).

### Statistical analysis

Data were evaluated for normality using Shapiro–Wilks tests and were transformed to ranks when not normally distributed. Student's *t*-test was used for comparisons between two groups, and one-way ANOVA was used for comparisons among more than two groups. Sequential follicle and hormone data across the cycle were analyzed using repeated-measures ANOVA (SAS PROC MIXED Version 9.2; SAS Institute, Inc.). Tukey's multiple range tests were used for *post hoc* analyses. Data are presented as mean (± SEM), while categorical data are presented as percentages. Significant differences are indicated by *P* < 0.05.

## Results

### Growth profiles for anovulatory dominant follicles vs ovulatory follicles

**Figure 2:**
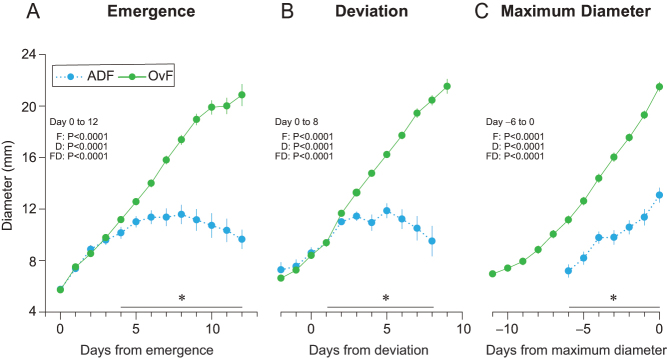
Mean (± SEM) diameter of anovulatory dominant follicles (ADF; *n* = 14) and ovulatory follicles (OvF; *n* = 49) within ovulatory cycles centralized to days of emergence, deviation, and maximum diameter. The probabilities for a follicle effect (F), day effect (D), and follicle-by-day interaction (FD) are shown. An asterisk (*) with a bar indicates days when differences (*P* < 0.05) in diameters between follicle types were observed.

Mean growth profiles for ADF and OvF, irrespective of wave, centralized to days of emergence, deviation, and maximum diameter are shown in Fig. 2A, B, and C. The mean diameters of ADF were smaller (*P* < 0.05) on the days of emergence, deviation, and maximum diameter compared to OvF.

### Intervals, diameters, and growth rates for anovulatory dominant follicles vs ovulatory follicles

Intervals between various follicular events, diameters, and growth rates for ADF vs OvF, irrespective of wave, are shown in Table 1. The interval from ovulation to emergence was shorter (*P* < 0.0001) for ADF than for OvF. The intervals from emergence to maximum diameter and deviation to maximum diameter were longer (*P* < 0.0001) for OvF than for ADF. The maximum diameter was greater (*P* < 0.0001) for OvF than for ADF. The growth rates from emergence and deviation to maximum diameter were greater (*P* < 0.0001) for OvF than for ADF. In addition, both OvF and ADF had faster (*P* < 0.0001) growth rates from deviation to maximum diameter than from emergence to deviation.

**Table 1:** Mean (± SEM) intervals, diameters, and growth rates of anovulatory dominant vs ovulatory follicles.

End point	ADF (*n* = 14)	OvF (*n* = 49)	*P*-value
Intervals (days)			
First ovulation to emergence^¥^	5.4 ± 2.0	16.2 ± 0.4	<0.0001
Emergence to deviation^§^	3.9 ± 0.6	3.4 ± 0.2	NS
Emergence to maximum diameter	6.7 ± 0.5	10.2 ± 0.2	<0.0001
Emergence to first day of regression/ovulation	8.5 ± 0.6	11.3 ± 0.2	<0.0001
Deviation to maximum diameter	2.9 ± 0.5	6.9 ± 0.2	<0.0001
Deviation to first day of regression/ovulation	4.6 ± 0.5	8.0 ± 0.2	<0.0001
Maximum diameter to first day of regression/ovulation	1.8 ± 0.2	1.2 ± 0.1	<0.0001
Diameters (mm)			
At emergence	5.7 ± 0.2	5.7 ± 0.1	NS
At deviation	9.4 ± 0.3	9.4 ± 0.2	NS
At maximum	13.1 ± 0.6	21.5 ± 0.3	<0.0001
Growth rates (mm/day)			
Emergence to deviation	1.1 ± 0.2^X^	1.2 ± 0.1^X^	NS
Emergence to maximum diameter	1.1 ± 0.1^X^	1.6 ± 0.0^Y^	<0.0001
Deviation to maximum diameter	1.4 ± 0.1^Y^	1.8 ± 0.0^Y^	<0.0001

ADF, anovulatory dominant follicle; NS, non-significant; OvF, ovulatory follicle.

^X,Y^Means with different superscripts within a column for the same end point are different (*P* < 0.05); ^¥^Day of emergence, day before the future dominant follicle reached 7 mm in diameter; ^§^Day of deviation, day the future dominant follicle started to grow at a faster rate than the largest subordinate follicle.

### Endocrine profiles associated with anovulatory dominant follicles vs ovulatory follicles

**Figure 3:**
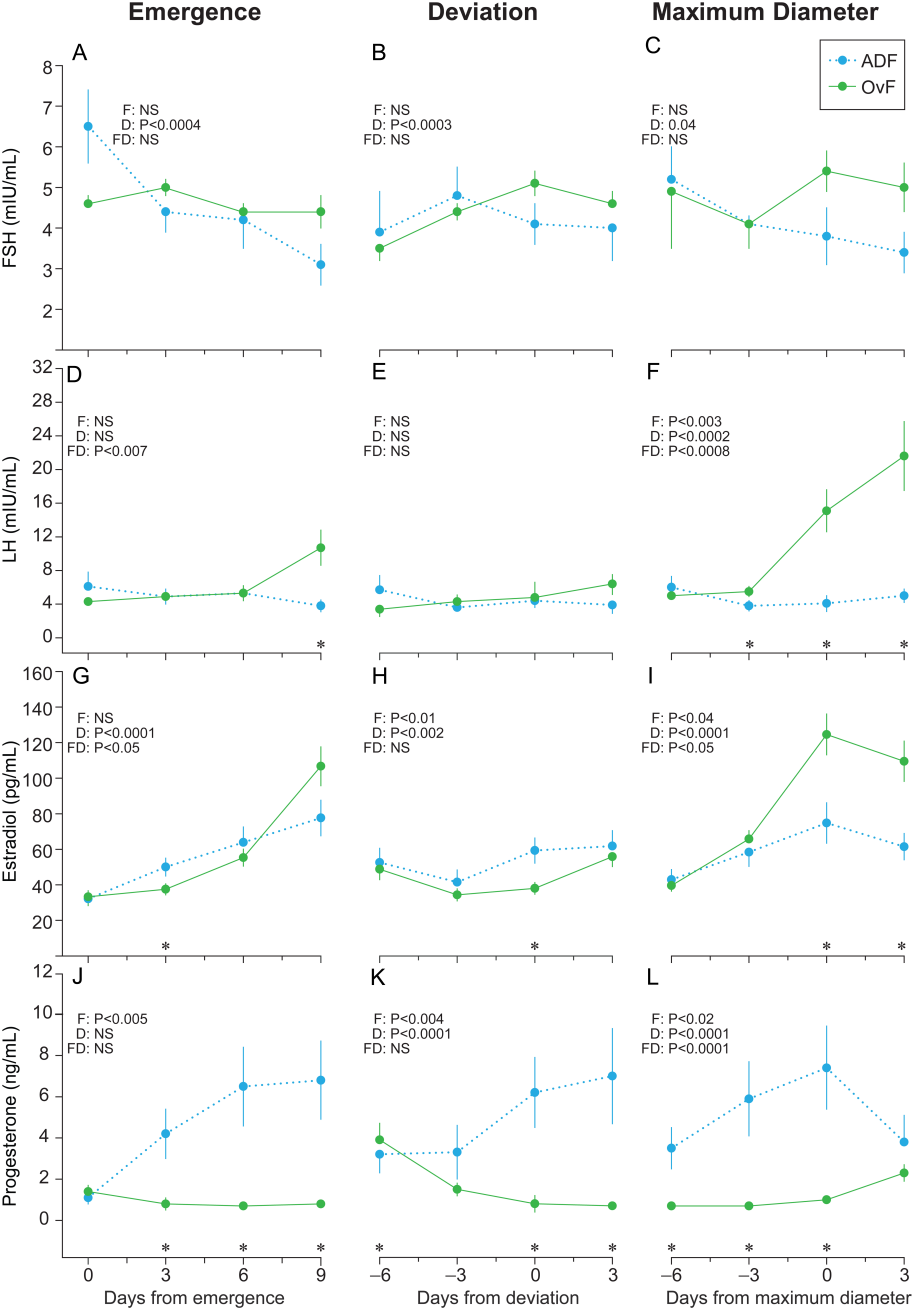
Mean (± SEM) concentrations of serum FSH, LH, estradiol, and progesterone associated with anovulatory dominant follicles (ADF; *n* = 14) and ovulatory follicles (OvF; *n* = 49) within ovulatory cycles centralized to days of follicle emergence, deviation, and maximum diameter. The probabilities for a follicle effect (F), day effect (D), and follicle-by-day interaction (FD) are shown. An asterisk (*) indicates days when differences (*P* < 0.05) between groups were observed.

Systemic hormone profiles were compared between OvF and ADF, irrespective of wave, after normalizing the data to the days of emergence, deviation, and maximum diameter (Fig. 3). An FSH decrease (*P* < 0.05) was first observed 6 days after emergence of ADF (Fig. 3A). FSH increased (*P* < 0.05) before deviation of OvF but did not increase in ADF (Fig. 3B). No difference between groups was observed for FSH before or after follicles reached maximum diameter (Fig. 3C).

LH increased (*P* < 0.05) 9 days after emergence of OvF; however, no increase was observed in ADF. LH concentrations associated with ADF and OvF did not change around the time of deviation (Fig. 3E). However, LH levels increased and were greater (*P* < 0.05) from 3 days before to 3 days after the maximum diameter of OvF compared to ADF (Fig. 3F).

Estradiol increased after emergence of ADF and OvF (Fig. 3G), after deviation of OvF (Fig. 3H), and before maximum diameter (Fig. 3I) of ADF and OvF. Progesterone was greater (*P* < 0.0001) after emergence (Fig. 3J), at and after deviation (Fig. 3K), and before and at the maximum diameter of ADF vs OvF (Fig. 3L). Progesterone increased (*P* < 0.05) 3 days after emergence of ADF but decreased 3 days after emergence of OvF.

Mean concentrations of hormones associated with follicle emergence, deviation, and maximum diameter of ADF vs OvF, irrespective of wave, are shown in Table 2. FSH was lower (*P* < 0.003) at emergence of OvF compared to ADF. Similarly, LH was lower (*P* < 0.004) at emergence of OvF when compared to ADF. LH was higher (*P* < 0.003) at maximum diameter of OvF vs ADF, but no differences at deviation were observed between groups. Although estradiol at emergence did not differ among groups, estradiol was greater (*P* < 0.0002) at deviation of ADF compared to OvF. However, estradiol was greater (*P* < 0.01) at maximum diameter of OvF compared to ADF. Progesterone did not differ between groups at emergence but was greater (*P* < 0.006) at deviation and maximum diameter of ADF in comparison to OvF. Maximum LH and estradiol concentrations were greater (*P* < 0.04) for OvF compared to ADF. Maximum and overall mean progesterone concentrations were lower (*P* < 0.0001) for OvF than for ADF.

**Table 2:** Mean (± SEM) hormone concentrations associated with various follicular events in anovulatory dominant vs ovulatory follicles.

End point	ADF (*n* = 14)	OvF (*n* = 49)	*P*-value
FSH (mIU/mL)			
Emergence^¥^	6.5 ± 0.9	4.6 ± 0.2	<0.003
Deviation^§^	4.1 ± 0.5	5.1 ± 0.3	<0.04
Maximum diameter	3.8 ± 0.7	5.1 ± 0.5	<0.07
LH (mIU/mL)			
Emergence	6.1 ± 1.7	4.3 ± 0.3^X^	<0.004
Deviation	4.4 ± 0.8	4.8 ± 0.4^X^	NS
Maximum diameter	4.1 ± 0.9	15.1 ± 2.5^Y^	<0.003
Estradiol (pg/mL)			
Emergence	32.1 ± 3.8^X^	33.3 ± 3.4^X^	NS
Deviation	54.8 ± 6.0^XY^	35.6 ± 2.3^X^	<0.0002
Maximum diameter	74.8 ± 11.4^Y^	122.0 ± 11.4^Y^	<0.01
Progesterone (ng/mL)			
Emergence	1.1 ± 0.2^X^	1.4 ± 0.3^X^	NS
Deviation	6.2 ± 1.7^XY^	0.8 ± 0.1^Y^	<0.006
Maximum diameter	7.4 ± 2.0^Y^	1.0 ± 0.1^X^	<0.0001
Maximum concentration			
FSH	5.4 ± 0.8	6.8 ± 0.4	NS
LH	7.5 ± 1.2	15.1 ± 2.1	<0.03
Estradiol	78.5 ± 10.8	130.9 ± 10.6	<0.04
Progesterone	7.9 ± 2.0	1.8 ± 0.2	<0.0001
Mean concentration^†^			
FSH	4.1 ± 0.5	4.8 ± 0.2	NS
LH	5.0 ± 0.7	7.0 ± 0.6	<0.05
Estradiol	62.3 ± 8.1	62.0 ± 3.5	NS
Progesterone	5.1 ± 1.3	1.0 ± 0.1	<0.0001

ADF, anovulatory dominant follicle; FSH, follicle stimulating hormone; LH, luteinizing hormone; NS, non-significant; OvF, ovulatory follicle.

^¥^Day of emergence, day before the future dominant follicle reached 7 mm in diameter; ^§^Day of deviation, day the future dominant follicle started to grow at a faster rate than the largest subordinate follicle; ^X,Y^Means with different superscripts within a column for the same end point are different (P < 0.05); ^†^Measured from the day of emergence to the day of maximum diameter of the dominant follicle.

### Growth profiles for W1ADF vs W2ADF, W2ADF vs W2OvF, and W2OvF vs W3OvF

**Figure 4:**
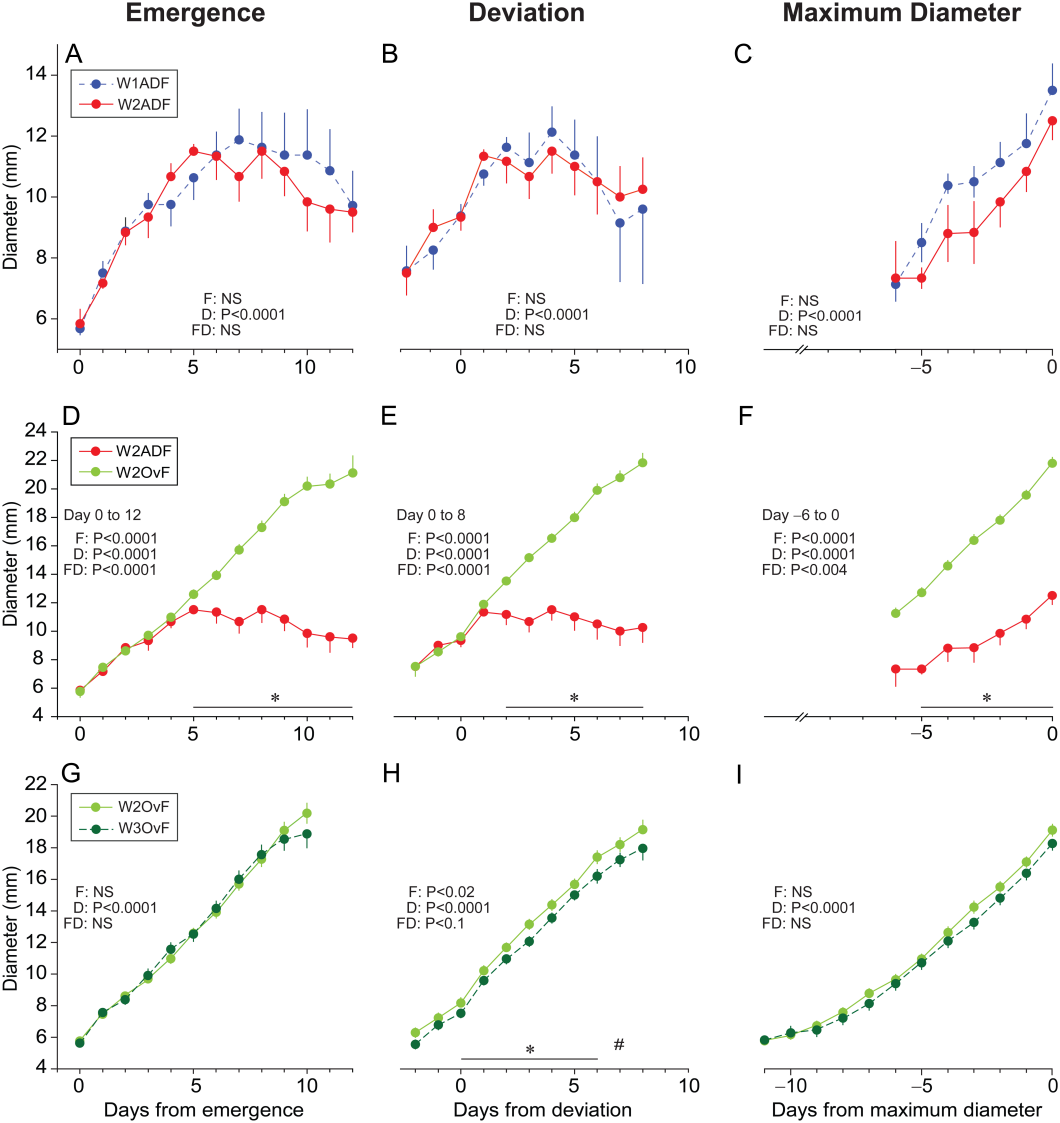
Mean (± SEM) diameters of (A, B, and C) wave 1 anovulatory dominant follicles (W1ADF; *n* = 8) vs wave 2 anovulatory dominant follicles (W2ADF; *n* = 6), (D, E, and F) wave 2 anovulatory dominant follicles (W2ADF; *n* = 6) vs wave 2 ovulatory follicles (W2OvF; *n* = 33), and (G, H, and I) wave 2 ovulatory follicles (W2OvF; *n* = 33) vs wave 3 ovulatory follicles (W3OvF; *n* = 16) centralized to days of follicle emergence, deviation, and maximum diameter. The probabilities for a follicle effect (F), day effect (D), and follicle-by-day interaction (FD) are shown. An asterisk (*) indicates days when differences (*P* < 0.05) in diameters between follicle types were observed, and a hash symbol (#) indicates differences that approached significance.

Follicle growth profiles centralized to the days of emergence, deviation, and maximum diameter of anovulatory or ovulatory waves are shown as follows: W1ADF (in women with two or three follicular waves) vs W2ADF (in women with three follicular waves; Fig. 4A, B, and C), W2ADF vs W2OvF (in women with two follicular waves; Fig. 4D, E, and F), and W2OvF vs W3OvF (in women with three follicular waves; Fig. 4G, H, and I). Follicle diameters did not differ between W1ADF and W2ADF. However, the mean diameter of W2ADF was smaller (*P* < 0.0001) than W2OvF when data were centralized to emergence, deviation, and maximum diameter. Moreover, the mean diameter of W2OvF was greater (*P* < 0.02) than W3OvF when data were centralized to deviation.

### Intervals, diameters, and growth rates for W1ADF vs W2ADF, W2ADF vs W2OvF, and W2OvF vs W3OvF

Intervals between various follicular events, diameters, and growth rates of anovulatory or ovulatory waves are shown as follows: W1ADF (in women with two or three follicular waves) vs W2ADF (in women with three follicular waves), and W2OvF (in women with two follicular waves) vs W3OvF (in women with three follicular waves; Table 3). The W1ADF emerged earlier (*P* < 0.0001) compared to W2ADF. The growth phase was longer (*P* < 0.02) for W1ADF vs W2ADF. In contrast, the regression phase was longer (*P* < 0.03) for W2ADF, even though the static phase was longer (*P* < 0.03) for W1ADF vs W2ADF. The mean follicle diameters and growth rates did not differ between W1ADF and W2ADF. A slower regression rate (*P* < 0.02) was observed for W2ADF. An earlier (*P* < 0.03) emergence relative to ovulation was observed for W2ADF compared to W2OvF, leading consequently to shorter intervals from emergence and deviation to the maximum diameter (Supplementary Table 1, see section on supplementary materials given at the end of this article). Moreover, the maximum diameter was greater (*P* < 0.0001) for W2OvF than for W2ADF. An earlier (*P* < 0.0001) emergence relative to the first ovulation was observed for W2OvF compared to W3OvF (Table 3); however, after emergence, the W2OvF took longer (*P* < 0.03) to ovulate than the W3OvF. The diameter of W3OvF at deviation was smaller (*P* < 0.02) when compared to W2OvF. Although the overall growth rates did not differ (*P* > 0.05) between W2OvF and W3OvF, the mean growth rates after deviation were greater (*P* < 0.02) in both W2OvF and W3OvF compared to those before deviation.

**Table 3:** Mean (± SEM) intervals, diameters, and growth rates in anovulatory dominant vs ovulatory follicles within and between different patterns of follicular waves.

End point	W1ADF (*n* = 8)	W2ADF (*n* = 6)	*P*-value	W2OvF (*n* = 33)	W3OvF (*n* = 16)	*P*-value
Intervals (days)						
First ovulation to emergence^¥^	**–**0.8 ± 0.5	13.5 ± 0.3	<0.0001	15.3 ± 0.4	18.3 ± 0.6	<0.0001
Emergence to deviation^§^	4.3 ± 1.0	3.3 ± 0.3	NS	3.6 ± 0.3	2.9 ± 0.3	<0.07
Emergence to maximum diameter	7.6 ± 0.6	5.5 ± 0.7	<0.02	10.5 ± 0.2	9.8 ± 0.4	<0.06
Emergence to first day of regression/ovulation	9.5 ± 0.8	7.2 ± 0.5	<0.02	11.6 ± 0.2	10.8 ± 0.4	<0.03
Emergence to regression at ≤7 mm	12.9 ± 1.1	13.8 ± 1.1	NS	**—**	**—**	—
Deviation to maximum diameter	3.4 ± 0.8	2.2 ± 0.7	NS	6.8 ± 0.3	6.9 ± 0.4	NS
Deviation to first day of regression/ovulation	5.3 ± 0.7	3.8 ± 0.7	<0.09	8.1 ± 0.3	7.9 ± 0.3	NS
Deviation to regression at ≤7 mm	8.6 ± 1.3	10.5 ± 1.3	NS	**—**	**—**	**—**
Maximum diameter to first day of regression/ovulation	1.8 ± 0.3	1.2 ± 0.2	<0.03	1.2 ± 0.1	1.1 ± 0.1	NS
Maximum diameter to regression at ≤7 mm	5.3 ± 0.9	8.3 ± 1.2	<0.03	**—**	**—**	**—**
Diameters (mm)						
At emergence	5.7 ± 0.2	5.8 ± 0.5	NS	5.8 ± 0.1	5.6 ± 0.2	NS
At deviation	9.4 ± 0.4	9.3 ± 0.4	NS	9.6 ± 0.2	8.9 ± 0.2	<0.02
At maximum	13.5 ± 0.9	12.5 ± 0.6	NS	21.8 ± 0.4	20.9 ± 0.6	<0.1
At Day –1	**—**	—		21.8 ± 0.4	20.8 ± 0.6	<0.09
Growth rates (mm/day)						
Emergence to deviation	0.9 ± 0.2^X^	1.2 ± 0.4	NS	1.2 ± 0.1^X^	1.3 ± 0.1^X^	NS
Emergence to maximum diameter	1.0 ± 0.1	1.3 ± 0.2	NS	1.6 ± 0.0	1.6 ± 0.1	NS
Deviation to maximum diameter	1.4 ± 0.2^Y^	1.5 ± 0.2	NS	1.8 ± 0.1^Y^	1.7 ± 0.1^Y^	NS
Deviation to Day –1	**—**	**—**		1.8 ± 0.1	1.7 ± 0.1	NS
Maximum diameter to regression at ≤7 mm	**–**1.6 ± 0.3	**–**0.8 ± 0.1	<0.02	**—**	**—**	**—**

NS, non-significant; W1ADF, wave 1 anovulatory dominant follicle; W2ADF, wave 2 anovulatory dominant follicle; W2OvF, wave 2 ovulatory follicle; W3OvF, wave 3 ovulatory follicle.

^¥^Day of emergence, day before the future dominant follicle reached 7 mm in diameter; ^§^Day of deviation, day the future dominant follicle started to grow at a faster rate than the largest subordinate follicle; ^X,Y^Means with different superscripts within a column for the same end point are different (*P* < 0.05).

### Endocrine profiles for W1ADF vs W2ADF, W2ADF vs W2OvF, and W2OvF vs W3OvF

**Figure 5:**
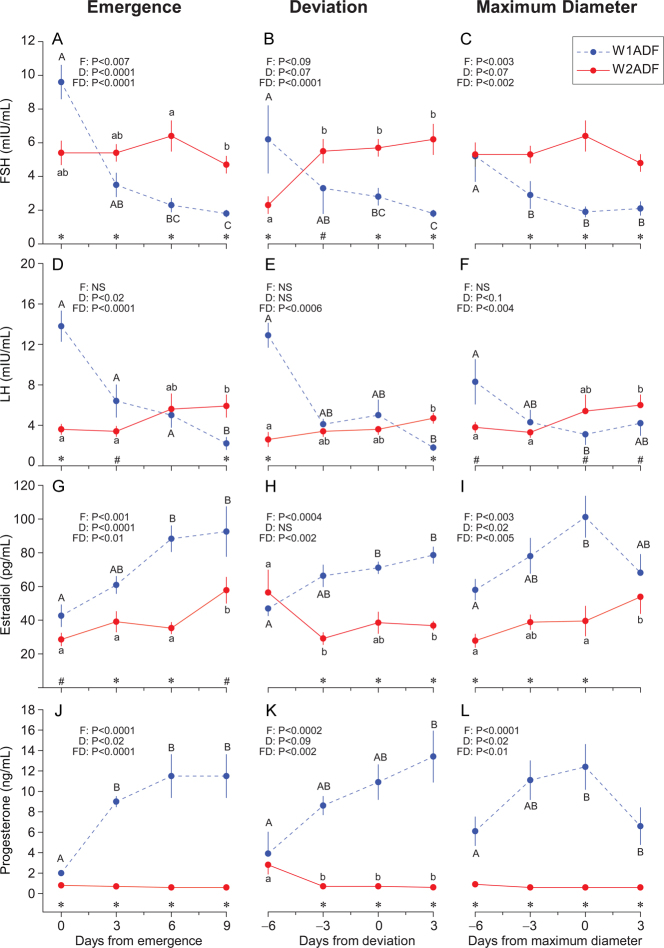
Mean (± SEM) concentrations of serum FSH, LH, estradiol, and progesterone associated with wave 1 anovulatory dominant follicles (W1ADF; *n* = 8) and wave 2 anovulatory dominant follicles (W2ADF; *n* = 6) within ovulatory cycles centralized to days of follicle emergence, deviation, and maximum diameter. Differences (*P* < 0.05) in hormone concentrations between time points are indicated by lowercase letters (a, b) for W2ADF and uppercase letters (A, B) for W1ADF. An asterisk (*) indicates days when differences (*P* < 0.05) between follicle types were observed, and a hash symbol (#) indicates differences that approached significance.

**Figure 6:**
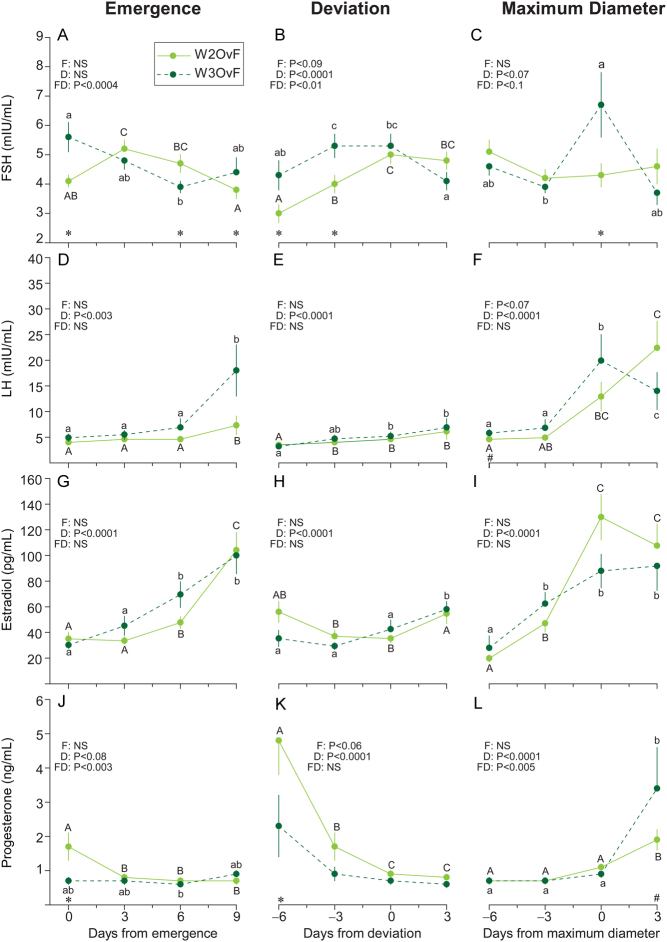
Mean (± SEM) concentrations of serum FSH, LH, estradiol, and progesterone associated with wave 2 ovulatory follicles (W2OvF; *n* = 33) and wave 3 ovulatory follicles (W3OvF; *n* = 16) centralized to days of follicle emergence, deviation, and maximum diameter. Differences (*P* < 0.05) in hormone concentrations between time points are indicated by lower-case letters (a, b) for W3OvF and upper-case letters (A, B) for W2OvF. An asterisk (*) indicates days when differences (*P* < 0.05) between groups were observed, and a hash symbol (#) indicates differences that approached significance.

FSH and LH consistently decreased after data were centralized to emergence (Fig. 5A and D), deviation (Fig. 5B and E), and maximum diameter (Fig. 5C and F) of W1ADF but not W2ADF. Estradiol and progesterone concentrations were greater (*P* < 0.003–*P* < 0.0002) when data were centralized to emergence, deviation, and maximum diameter (Fig. 5G, H, I, J, K, and L) of W1ADF compared to W2ADF. Hormone concentrations associated with W2ADF vs W2OvF are shown in Supplementary Fig. 1. Concentrations of FSH were higher (*P* < 0.05) at emergence of W2ADF vs W2OvF, and the overall concentrations of estradiol were lower (*P* < 0.0001) when normalized to maximum diameter of W2ADF than W2OvF. Overall, concentrations of FSH, LH, estradiol, and progesterone did not differ (*P* > 0.05) when data were centralized to emergence, deviation, and maximum diameter of W2OvF vs W3OvF (Fig. 6A, B, C, D, E, F, G, H, I, J, K, and L). However, concentrations of FSH were higher (*P* < 0.05) at emergence of W3OvF than W2OvF.

Mean concentrations of hormones at follicle emergence, deviation, and maximum diameter of W1ADF vs W2ADF, W2ADF vs W2OvF, and W2OvF vs W3OvF are shown in Table 4 and Supplementary Table 2. At emergence, FSH and LH concentrations were greater (*P* < 0.003) for W1ADF than for W2ADF. In contrast, at deviation and maximum diameter, FSH was greater (*P* < 0.0002) for W2ADF compared to W1ADF. Estradiol and progesterone were greater (*P* < 0.03) at emergence, deviation, and maximum diameter of W1ADF vs W2ADF. Similarly, maximum and mean concentrations of estradiol and progesterone from emergence to maximum diameter were greater (*P* < 0.0003) for W1ADF than for W2ADF. FSH concentrations were greater (*P* < 0.05) at emergence and maximum diameter of W2ADF vs W2OvF (Supplementary Table 2). Estradiol and progesterone were greater (*P* < 0.04) at maximum diameter and maximum concentration of W2OvF than W2ADF. Moreover, the mean concentrations of LH, estradiol, and progesterone were greater (*P* < 0.01–0.09) from emergence to maximum diameter for W2OvF compared to W2ADF. Greater (*P* < 0.01) FSH concentrations were observed at emergence, maximum diameter, and ovulation in W3OvF compared to W2OvF (Table 4). At ovulation, LH was greater (*P* < 0.04) in W3OvF compared to W2OvF. Mean FSH and LH were greater (*P* < 0.03–0.08) from emergence to maximum diameter of W3OvF vs W2OvF. Progesterone concentrations at emergence and from emergence to maximum diameter were greater (*P* < 0.05) in W2OvF compared to W3OvF.

**Table 4:** Mean (± SEM) hormone concentrations associated with anovulatory dominant vs ovulatory follicles within and between different patterns of follicular waves.

End point	W1ADF (*n* = 8)	W2ADF (*n* = 6)	*P*-value	W2OvF (*n* = 33)	W3OvF (*n* = 16)	*P*-value
FSH (mIU/mL)						
Emergence^¥^	9.6 ± 1.0^X^	5.4 ± 0.7	<0.003	4.1 ± 0.2	5.6 ± 0.5	<0.001
Deviation^§^	2.8 ± 0.5^Y^	5.7 ± 0.5	<0.0002	5.0 ± 0.3	5.3 ± 0.4	NS
Maximum diameter	1.9 ± 0.3^Y^	6.4 ± 0.9	<0.0001	4.3 ± 0.4	6.7 ± 1.1	<0.01
Ovulation	—	—		4.1 ± 0.5	6.3 ± 1.1	<0.02
LH (mIU/mL)						
Emergence	13.8 ± 1.5^X^	3.6 ± 0.5	<0.0001	4.0 ± 0.4^X^	4.9 ± 0.7^X^	<0.1
Deviation	5.0 ± 1.5^Y^	3.6 ± 0.5	NS	4.6 ± 0.4^X^	5.2 ± 0.8^X^	NS
Maximum diameter	3.1 ± 1.0^Y^	5.4 ± 1.6	<0.07	12.9 ± 2.8^Y^	19.9 ± 5.1^Y^	<0.1
Ovulation	—	—		12.6 ± 3.0^Y^	23.3 ± 6.2^Y^	<0.04
Estradiol (pg/mL)						
Emergence	42.7 ± 6.4^X^	28.6 ± 3.8	<0.03	35.0 ± 5.0^X^	30.1 ± 2.5^X^	NS
Deviation	71.1 ± 3.4^XY^	38.5 ± 6.3	<0.0002	35.3 ± 3.3^X^	36.0 ± 3.0^X^	NS
Maximum diameter	101.2 ± 12.2^Y^	39.5 ± 8.8	<0.001	133.6 ± 15.5^Y^	96.9 ± 11.4^Y^	<0.07
Ovulation	—	—		111.0 ± 11.3^Y^	133.0 ± 19.8^Y^	NS
Progesterone (ng/mL)						
Emergence	2.0 ± 0.0^X^	0.8 ± 0.1	<0.0001	1.7 ± 0.4^X^	0.7 ± 0.1	<0.03
Deviation	10.9 ± 1.7^Y^	0.7 ± 0.1	<0.0001	0.9 ± 0.1^Y^	0.6 ± 0.1	<0.06
Maximum diameter	12.4 ± 2.2^Y^	0.6 ± 0.1	<0.0002	1.1 ± 0.1^XY^	0.9 ± 0.1	NS
Ovulation	—	—		1.3 ± 0.2^XY^	1.3 ± 0.3	NS
Maximum concentration						
FSH	9.6 ± 1.0	6.6 ± 0.8	<0.1	6.5 ± 0.5	7.6 ± 0.8	<0.09
LH	8.9 ± 1.6	5.8 ± 1.5	NS	13.0 ± 2.4	19.9 ± 5.1	<0.08
Estradiol	104.4 ± 11.2	44.0 ± 7.9	<0.0008	131.8 ± 14.2	129.2 ± 15.1	NS
Progesterone	13.3 ± 1.9	0.8 ± 0.1	<0.0001	2.0 ± 0.4	1.2 ± 0.2	<0.06
Mean concentration^†^						
FSH	3.1 ± 0.6	5.6 ± 0.6	<0.008	4.6 ± 0.2	5.1 ± 0.3	<0.08
LH	5.7 ± 1.1	4.1 ± 0.8	NS	6.2 ± 0.6	8.6 ± 1.3	<0.03
Estradiol	82.6 ± 7.8	35.2 ± 5.2	<0.0003	60.5 ± 4.4	65.2 ± 5.5	NS
Progesterone	8.3 ± 1.4	0.7 ± 0.1	<0.0002	1.1 ± 0.1	0.8 ± 0.1	<0.05

NS, non-significant; W1ADF, wave 1 anovulatory dominant follicle; W2ADF, wave 2 anovulatory dominant follicle; W2OvF, wave 2 ovulatory follicle; W3OvF, wave 3 ovulatory follicle.

^¥^Day of emergence, day before the future dominant follicle reached 7 mm in diameter; ^§^Day of deviation, day the future dominant follicle started to grow at a faster rate than the largest subordinate follicle; ^X,Y^Means with different superscripts within a column for the same end point are different (*P* < 0.05); ^†^Measured from the day of emergence to the day of maximum diameter of the dominant follicle.

## Discussion

To our knowledge, this is the first comprehensive evaluation of intervals, growth rates, diameters, and associated systemic endocrine profiles of ADF and OvF that developed from different waves (i.e., W1ADF, W2ADF, W2OvF, and W3OvF) within and between menstrual cycles. From these analyses, we were able to document differences in growth and endocrine profiles between ADF and OvF, as well as between the different types of anovulatory vs ovulatory waves.

### Growth profiles and intervals of W1ADF vs W2ADF, W2ADF vs W2OvF, and W2OvF vs W3OvF

This study provides insight into the growth dynamics of anovulatory and ovulatory follicles developing in spontaneous ovulatory menstrual cycles. However, because ADF and OvF emerged at different times from the preceding ovulation (luteal vs follicular phase), differences in endocrine profiles were expected based on the presence or absence of corpus luteum (Ginther *et al.* 1989, 2005, Baerwald 2009). Therefore, division into the type of follicular wave was undertaken to better understand potential differences between the types of anovulatory and ovulatory waves.

Within ovulatory cycles, W1ADF (in women with two or three follicular waves) grew for a longer period of time than W2ADF (in women with three follicular waves). This finding supports results from earlier reports demonstrating shorter inter-wave intervals for three- vs two-wave cycles in women and heifers (Ginther *et al.* 1989, Baerwald *et al.* 2003*b*). Furthermore, W1ADF had a longer static phase than W2ADF. The regression phase of W2ADF was longer compared to W1ADF; this outcome complements a separate study that showed that follicles regressed faster in women with three vs two follicular waves (Baerwald* et al.* 2009). Another important observation was a shorter growth phase and smaller diameter of ovulatory follicles from emergence to ovulation in W3OvF (in women with three follicular waves) than in W2OvF (in women with two follicular waves). Interestingly, W2ADF (in women with three follicular waves) emerged earlier compared to W2OvF (in women with two follicular waves), indicating that timing of emergence relative to the luteal phase impacts the outcome of the follicle. The potential impact of luteal function on the development of anovulatory vs ovulatory follicular waves is described below. Studies in heifers have shown that preovulatory follicles of the third wave have a smaller diameter and a shorter interval from emergence to ovulation (Sirois & Fortune 1988, Ginther *et al.* 1989, 2013*c*). We interpreted these results to indicate the potential need for a shorter growth phase and smaller diameter of ovulatory follicles in three- vs two-wave cycles to potentially keep the IOI within the normal range for the species. In addition, the findings further validate similarities in antral follicular wave dynamics among humans and livestock species.

An increased growth rate after deviation was observed in W1ADF (in women with two or three follicular waves) compared to W2ADF (in women with three follicular waves). Although growth rates did not differ between W1ADF and W2ADF, W2ADF and W2OvF, or W2OvF and W3OvF, W1ADF regressed more quickly compared to W2ADF. Differences in growth rates of dominant follicles between anovulatory and ovulatory waves were not reported in the original study (Baerwald *et al.* 2003*a*), and data were not partitioned into first vs second anovulatory waves or ovulatory waves of the IOI. In considering the purpose of follicular waves in women, we propose that anovulatory follicular waves develop throughout the luteal and early follicular phases to provide a wave of follicles ready and capable of undergoing selection and ovulation if provided with the proper hormonal milieu. In women with either two or three waves, it is considered that luteal phase-dominant follicles are anovulatory due to luteal progesterone production, which inhibits the LH surge and thereby inhibits ovulation. In women with three waves, the first wave of the follicular phase (which is typically the second wave of the IOI) results in the development of an anovulatory-dominant follicle. The reason this wave does not become ovulatory is not understood, but luteal progesterone may, again, be involved. As soon as the largest follicle of the second wave (W2ADF) of the IOI regresses, a third wave (W3OvF) develops, resulting in ovulation.

Increases in follicle growth rates after deviation in our study are consistent with those reported previously in the human and equine species (Ginther *et al.* 2004, Baerwald* et al.* 2009). Greater follicle growth rates have been reported for minor waves compared to major waves (Baerwald *et al.* 2003*b*); minor waves were not evaluated in the present study. Future studies focused on the follicular fluid endocrine milieu, proteomics, and extracellular vesicle-coupled microRNA dynamics, as well as on granulosa cell gene expression in W1ADF and W2ADF vs W2OvF and W3OvF, as recently performed in mares during different phases of the ovulatory wave (Wischral *et al.* 2021, Ishak *et al.* 2022, Gebremedhn *et al.* 2023), would provide insight into why major anovulatory follicle waves develop prior to ovulation in women. Recently, a follicle wall biopsy (FWB) technique was developed in our lab (Ishak *et al.* 2018) to study in mares the expression pattern of growth factors and hormone receptors and gene expression in the layers (i.e., granulosa and theca) of the follicular wall (Ishak *et al.* 2019, 2020) in association with the follicular fluid milieu. Therefore, the FWB technique might become a tool to be explored in women to identify cellular and molecular differences between anovulatory and ovulatory follicles.

### Endocrine profiles of W1ADF vs W2ADF, W2ADF vs W2OvF, and W2OvF vs W3OvF

Further evaluation of hormonal profiles between W1ADF (in women with two or three waves) and W2ADF (in women with three waves) revealed that FSH and LH concentrations decreased throughout W1ADF but were consistently higher in W2ADF. Decreasing FSH concentrations after emergence and before and after deviation of the future ovulatory follicle has been reported previously in women (Ginther *et al.* 2005). In addition, W1ADF were associated with greater levels of serum estradiol and progesterone compared to W2ADF. Progesterone and inhibin produced by the corpus luteum have been shown to have a negative feedback effect on the hypothalamus–pituitary axis, resulting in lower concentrations of circulating gonadotropins (Yamoto *et al.* 1991, Skinner *et al.* 1998, Sleiter *et al.* 2009). The findings from the present study support this notion, as overall, W1ADF grew in association with higher progesterone and lower FSH compared to W2ADF simply because W1ADF emerged sooner in the luteal phase when the corpus luteum was functional. We have more recently obtained preliminary data to demonstrate further that inhibin A and B are lower in women that develop ADFs in the luteal phase of the cycle (Vanden Brink *et al.* 2015). Higher levels of estradiol in association with W1ADF were partly due to the production of estradiol from the corpus luteum since W1ADF emerged around the day of ovulation in women. The presence of steroidogenic enzymes, as well as the secretion of estradiol, has been reported in human luteal tissue *in vitro* (Devoto *et al.* 2009). A prolonged elevation in luteal phase estradiol has been reported previously in women of reproductive age with three vs two follicular waves (Baerwald *et al.* 2003*b*). Furthermore, a greater serum concentration of luteal phase estradiol has been documented in women of advanced reproductive age developing luteal phase-dominant follicles (Vanden Brink *et al.* 2015). In both cases, elevated and prolonged luteal phase estradiol was attributed to the simultaneous growth of anovulatory follicles and the corpus luteum. When distinguishing between minor and major anovulatory waves during the luteal phase in women of reproductive age, no differences in serum estradiol were found (Ginther *et al.* 2005). Additional research is required to confirm a luteal vs follicular origin of luteal phase estradiol production in women with minor vs major follicular waves across the reproductive lifespan.

Low estradiol and progesterone concentrations with no increase after selection of W2ADF occurred in association with luteal regression, suggesting that a significant complement of luteal phase estradiol originates from the corpus luteum in at least some women. Some luteal phase-dominant follicles have been shown to be estrogenic while others are not (Baerwald *et al.* 2003*b*, Vanden Brink *et al.* 2015). Follicular phase estradiol concentrations have been low in women who were classified as poor responders to ovarian stimulation (de los Santos *et al.* 2013). Recent studies have shown that initiation of ovarian stimulation in the luteal phase or randomly throughout the cycle in women with a history or prediction of a poor response to stimulation results in similar oocyte retrieval, blastocyst production, pregnancy, and live birth rates (for review, seeBaerwald & Pierson 2020). These clinical findings demonstrate comparable developmental competence of oocytes from estrogenic-dominant follicles developing within luteal vs follicular phase waves.

Another intriguing observation was the increase in serum FSH at maximum follicle diameter of W2ADF and W3OvF in women with three follicular waves but not W2OvF in women with two waves. The greater FSH at maximum diameter of W2ADF was due to the early follicular phase rise in FSH, which was responsible for inducing its emergence. The greater FSH at maximum diameter of W3OvF was attributed to the late follicular phase preovulatory FSH surge. In cattle, a minor (i.e., secondary) FSH surge has been detected prior to the preovulatory FSH surge in association with the emergence of the first luteal phase follicular wave (Ginther *et al.* 2013*b*, 2014). Secondary FSH surges have not been characterized in women to date due to the impracticability of frequent (e.g., hourly) blood sampling. Higher mean FSH during the IOI has been hypothesized as a plausible cause for the occurrence of three vs two follicular waves in heifers (Ginther *et al.* 2013*a*). In the current study, W3OvF were associated with greater levels of FSH and LH at emergence, maximum diameter, and ovulation and lower progesterone at emergence and deviation compared to W2OvF. Therefore, perhaps the corpus luteum activity associated with higher circulating concentrations of progesterone and inhibin may regulate follicle emergence in W2OvF. Future studies should focus on obtaining frequent blood samples to characterize episodic increases in FSH associated with wave emergence throughout the cycle in women.

### Comparison with previous studies, limitations, and future directions

Earlier studies (Baerwald *et al.* 2003*a*,* b *, 2005, Ginther *et al.* 2004, 2005) characterizing antral follicle dynamics and endocrine profiling have provided insight into understanding folliculogenesis during spontaneous ovulatory menstrual cycles in women. In the present study, we detected differences in growth and endocrine profiles between ADF and OvF as well as between W1ADF and W2ADF, W2ADF and W2OvF, and W2OvF and W3OvF within spontaneous ovulatory cycles. Evaluating mean hormone profiles associated with anovulatory vs ovulatory follicles developing within different waves of the IOI provided novel insight into understanding hormonal mechanisms underlying follicular waves.

The biggest limitation in our analysis was that a 3-day blood-sampling interval did not allow characterization of more frequent changes in hormone production during the IOI. As a result, it was difficult to develop a clear understanding of the causation of two- vs three-follicular wave cycles. Preliminary findings from our initial studies (Baerwald *et al.* 2003*b*) showed that FSH began to rise 3 days prior to wave emergence. In the present study, only data on the day of emergence and later were shown. Therefore, changes in FSH preceding wave emergence were not captured. We are currently conducting additional research with daily blood sampling across the menstrual cycle to shed further light on the hormonal regulation of ovarian follicular wave dynamics in women. Future studies should investigate the occurrence of a minor/secondary FSH surge before ovulation and its role in inducing the emergence of the luteal phase wave, as previously shown in the bovine model (Ginther *et al.* 2013*b*). Another limitation of the present study was that W1ADF developing from either a two- or three-wave cycle were combined. Evaluating W1ADF of two- vs three-wave cycles separately would have provided additional insight to understand the mechanisms underlying the development of two vs three follicular waves.

Understanding the relationship between the type of follicular wave and the respective quality of oocytes is vital to further improve the efficiency of ovarian stimulation protocols, the production of embryos *in vitro*, and the overall live birth rates after assisted reproductive technologies (ART) (Lin *et al.* 2018, İsrafilova *et al.* 2021, Balkenende *et al.* 2022). Therefore, greater knowledge about hormonal regulation of anovulatory and ovulatory follicular waves throughout the menstrual cycle is fundamental for understanding how to effectively suppress follicular growth and/or ovulation with hormonal contraceptive therapies. The clinical applications of follicular waves to date have been most apparent for women requiring urgent ovarian stimulation and oocyte retrieval prior to chemotherapy as well as women requiring double stimulation due to an initial poor response (Baerwald & Pierson 2020). However, further knowledge about how pituitary, follicular, and luteal hormones regulate follicular waves in women will allow continued optimization of ovarian stimulation strategies (Lin *et al.* 2018, İsrafilova *et al.* 2021, Balkenende *et al.* 2022); this will facilitate the comprehension of the influence of the corpus luteum during the luteal phase or random-start ovarian stimulation protocols. In addition, a greater understanding of hormonal regulation of anovulatory vs ovulatory follicular waves may provide further insight into developing novel strategies for allowing dominant follicle growth but preventing ovulation in future hormonal contraceptive formulations.

## Conclusions

In summary, progressively decreasing gonadotropins, slower growth rates, longer growth phases, and more rapid regression rates were observed for W1ADF (in women with a two- or three-wave cycle) compared to W2ADF (in women with three waves). FSH was greater and estradiol lower in W2ADF compared to W1ADF. W2OvF in women with two waves was associated with lower mean FSH and higher estradiol compared to women who had W2ADF in a three-wave cycle. Late luteal phase-early follicular phase emergence of W2ADF results in regression, enabling the emergence of a third follicular wave in the cycle. Greater serum FSH concentrations were detected in association with the growth of W3OvF compared to W2OvF. The data were interpreted to mean that there is a potential role of FSH in the development of three vs two follicular waves. Collectively, this knowledge may be applied to the future optimization of ovarian stimulation protocols for assisted reproduction and hormonal contraceptive formulations and provide directions for future studies to elucidate the physiologic mechanisms underlying selection of the dominant follicle, ovulation, and pathophysiology of anovulation in women.

## Supplementary Materials

Supplementary Figure S1

Supplementary Table S1. Mean (± SEM) intervals, diameters, and growth rates associated with wave 2 anovulatory dominant versus ovulatory follicles.

Supplementary Table S2. Mean (± SEM) hormone concentrations associated with various follicular events in wave 2 anovulatory dominant versus ovulatory follicles.

## Declaration of interest

The authors declare that there is no conflict of interest that could be perceived as prejudicing the impartiality of the research reported.

## Funding

No external funding sources were used for this study. Funding for the original study from which the data were obtained was provided by an operating grant from the Canadian Institutes for Health Researchhttp://dx.doi.org/10.13039/100005622. Trial registration number: ClinicalTrials.gov Identifier: NCT01389141.

## Data availability

The data underlying this article are available in the article and in its online supplementary material.

## Author contribution statement

S T B and E L G conceived the study, analyzed and interpreted data, and wrote the manuscript. M O G helped with data processing, statistical analyses, and manuscript preparation. A R B and R A P helped to plan the study and participated in data collection, interpretation, and manuscript revision. All the authors critically reviewed the manuscript.
