# Supplementary figures and images for: Dominant follicle growth patterns and associated endocrine dynamics in anovulatory and ovulatory waves in women
Source: Reprod Fertil. 2023 Jun 21;4(2):e220131. doi: 10.1530/RAF-22-0131 (PMC10305562; doi:10.1530/RAF-22-0131)

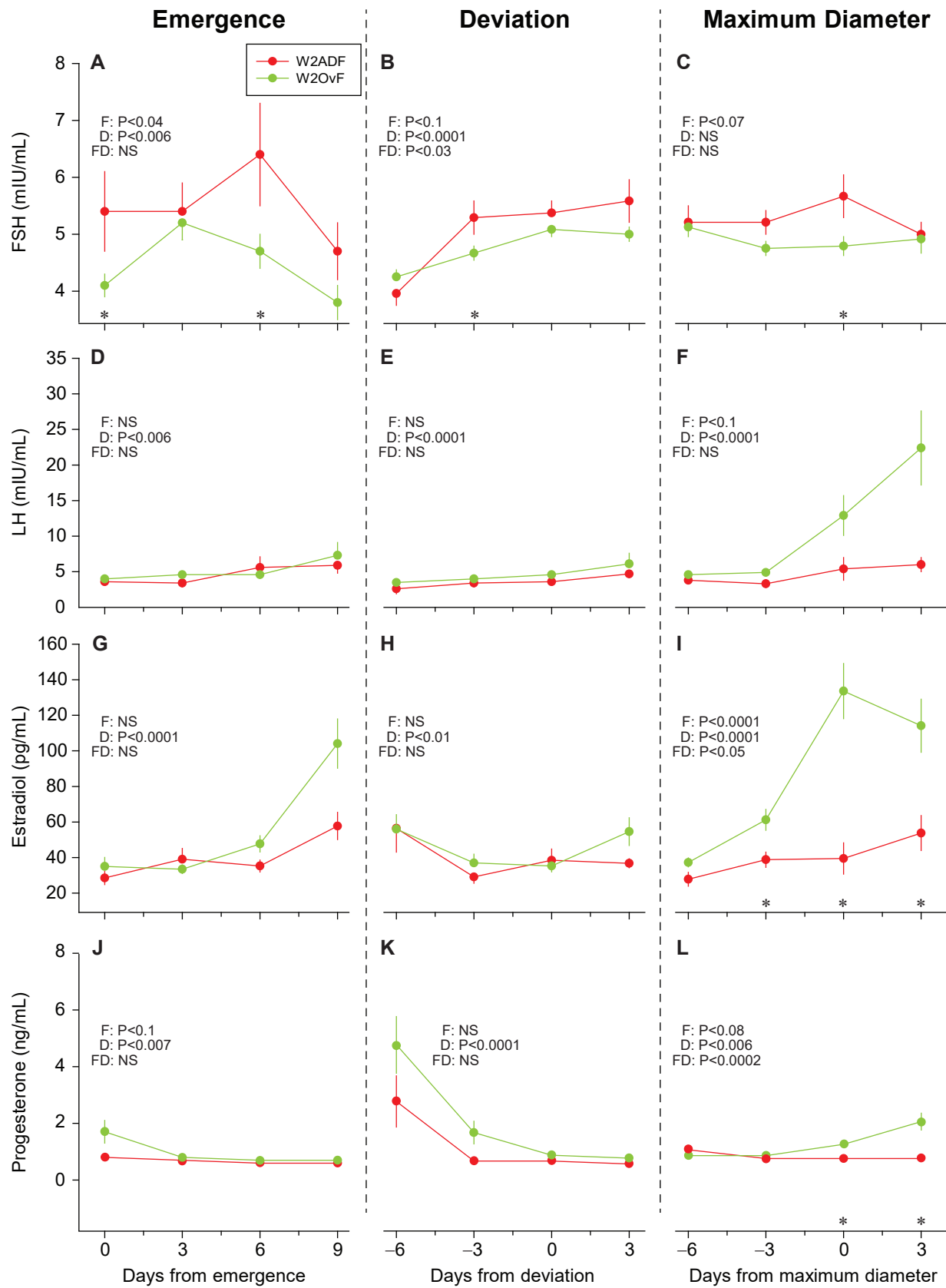

Suppl. Fig. 1

Supplement: Supplementary Figure S1 [file supplementary_figure_1.pdf]
