# Supplementary material for: Dominant follicle growth patterns and associated endocrine dynamics in anovulatory and ovulatory waves in women
Source: Reprod Fertil. 2023 Jun 21;4(2):e220131. doi: 10.1530/RAF-22-0131 (PMC10305562; doi:10.1530/RAF-22-0131)
Supplement: Supplementary Table S1. Mean (± SEM) intervals, diameters, and growth rates associated with wave 2 anovulatory dominant versus ovulatory follicles. [file supplementary_table_1.pdf]

Supplementary Table S1. Mean (± SEM) intervals, diameters, and growth rates associated with wave 2 anovulatory dominant versus ovulatory follicles.

**End point**	**W2ADF (*n* = 6)**	**W2OvF (*n* = 33)**	***P*-value**
**Intervals (days)**			
First ovulation to emergence[¥]	13.5 ± 0.3	15.3 ± 0.4	<0.03
Emergence to deviation[§]	3.3 ± 0.3	3.6 ± 0.3	NS
Emergence to maximum diameter	5.5 ± 0.7	10.5 ± 0.2	<0.0001
Emergence to first day of regression/ovulation	7.2 ± 0.5	11.6 ± 0.2	<0.0001
Deviation to maximum diameter	2.2 ± 0.7	6.8 ± 0.3	<0.0001
Deviation to first day of regression/ovulation	3.8 ± 0.7	8.1 ± 0.3	<0.0001
Maximum diameter to first day of regression/ovulation	1.2 ± 0.2	1.2 ± 0.1	NS
**Diameters (mm)**			
At emergence	5.8 ± 0.5	5.8 ± 0.1	NS
At deviation	9.3 ± 0.4	9.6 ± 0.2	NS
At maximum	12.5 ± 0.6	21.8 ± 0.4	<0.0001
**Growth rates (mm/day)**			
Emergence to deviation	1.2 ± 0.4	1.2 ± 0.1	NS
Emergence to maximum diameter	1.3 ± 0.2	1.6 ± 0.0	NS
Deviation to maximum diameter	1.5 ± 0.2	1.8 ± 0.1	NS

W2ADF, wave 2 anovulatory dominant follicle; W2OvF, wave 2 ovulatory follicle; NS, non-significant.
[¥]Day of emergence, day before the future dominant follicle reached 7 mm in diameter.
[§]Day of deviation, day the future dominant follicle started to grow at a faster rate than the largest subordinate follicle.
