# Supplementary material for: Dominant follicle growth patterns and associated endocrine dynamics in anovulatory and ovulatory waves in women
Source: Reprod Fertil. 2023 Jun 21;4(2):e220131. doi: 10.1530/RAF-22-0131 (PMC10305562; doi:10.1530/RAF-22-0131)
Supplement: Supplementary Table S2. Mean (± SEM) hormone concentrations associated with various follicular events in wave 2 anovulatory dominant versus ovulatory follicles. [file supplementary_table_2.pdf]

Supplementary Table S2. Mean (± SEM) hormone concentrations associated with various follicular events in wave 2 anovulatory dominant versus ovulatory follicles.

**End point**	**W2ADF (*n* = 6)**	**W2OvF (*n* = 33)**	***P*-value**
**FSH (mIU/mL)**			
Emergence[¥]	5.4 ± 0.7	4.1 ± 0.2	<0.05
Deviation[§]	5.7 ± 0.5	5.0 ± 0.3	NS
Maximum diameter	6.4 ± 0.9	4.3 ± 0.4	<0.02
**LH (mIU/mL)**			
Emergence	3.6 ± 0.5	4.0 ± 0.4^{X}	NS
Deviation	3.6 ± 0.5	4.6 ± 0.4^{X}	NS
Maximum diameter	5.4 ± 1.6	12.9 ± 2.8^{Y}	NS
**Estradiol (pg/mL)**			
Emergence	28.6 ± 3.8	35.0 ± 5.0^{X}	NS
Deviation	38.5 ± 6.3	35.3 ± 3.3^{X}	NS
Maximum diameter	39.5 ± 8.8	133.6 ± 15.5^{Y}	<0.0001
**Progesterone (ng/mL)**			
Emergence	0.8 ± 0.1	1.7 ± 0.4^{X}	NS
Deviation	0.7 ± 0.1	0.9 ± 0.1^{Y}	NS
Maximum diameter	0.6 ± 0.1	1.1 ± 0.1^{XY}	<0.04
**Maximum concentration**			
FSH	6.6 ± 0.8	4.3 ± 0.5	<0.03
LH	5.8 ± 1.5	13.0 ± 2.4	NS
Estradiol	44.0 ± 7.9	131.8 ± 14.2	<0.005
Progesterone	0.8 ± 0.1	2.0 ± 0.4	<0.08
**Mean concentration**[†]			
FSH	5.6 ± 0.6	4.6 ± 0.2	<0.05
LH	4.1 ± 0.8	6.2 ± 0.6	<0.09
Estradiol	35.2 ± 5.2	60.5 ± 4.4	<0.01
Progesterone	0.7 ± 0.1	1.1 ± 0.1	<0.07

W2ADF, wave 2 anovulatory dominant follicle; W2OvF, wave 2 ovulatory follicle; NS, non-significant.

[¥]Day of emergence, day before the future dominant follicle reached 7 mm in diameter.

[§]Day of deviation, day the future dominant follicle started to grow at a faster rate than the largest subordinate follicle.

^{X,Y}Means with different superscripts within follicle class are different ($P < 0.05$).

[†]Measured from the day of emergence to the day of maximum diameter of the dominant follicle.
